# MLAGO: machine learning-aided global optimization for Michaelis constant estimation of kinetic modeling

**DOI:** 10.1186/s12859-022-05009-x

**Published:** 2022-11-01

**Authors:** Kazuhiro Maeda, Aoi Hatae, Yukie Sakai, Fred C. Boogerd, Hiroyuki Kurata

**Affiliations:** 1 Department of Bioscience and Bioinformatics, Kyushu Institute of Technology, 680-4 Kawazu, Iizuka, Fukuoka 820-8502 Japan; 2 Department of Molecular Cell Biology, Faculty of Science, VU University Amsterdam, O|2 Building, Amsterdam, The Netherlands

**Keywords:** Simulation, Michaelis constant, Kinetic modeling, Parameter estimation, Machine learning, Global optimization, Systems biology

## Abstract

**Background:**

Kinetic modeling is a powerful tool for understanding the dynamic behavior of biochemical systems. For kinetic modeling, determination of a number of kinetic parameters, such as the Michaelis constant (K_m_), is necessary, and global optimization algorithms have long been used for parameter estimation. However, the conventional global optimization approach has three problems: (i) It is computationally demanding. (ii) It often yields unrealistic parameter values because it simply seeks a better model fitting to experimentally observed behaviors. (iii) It has difficulty in identifying a unique solution because multiple parameter sets can allow a kinetic model to fit experimental data equally well (the non-identifiability problem).

**Results:**

To solve these problems, we propose the Machine Learning-Aided Global Optimization (MLAGO) method for K_m_ estimation of kinetic modeling. First, we use a machine learning-based K_m_ predictor based only on three factors: EC number, KEGG Compound ID, and Organism ID, then conduct a constrained global optimization-based parameter estimation by using the machine learning-predicted K_m_ values as the reference values. The machine learning model achieved relatively good prediction scores: RMSE = 0.795 and R^2^ = 0.536, making the subsequent global optimization easy and practical. The MLAGO approach reduced the error between simulation and experimental data while keeping K_m_ values close to the machine learning-predicted values. As a result, the MLAGO approach successfully estimated K_m_ values with less computational cost than the conventional method. Moreover, the MLAGO approach uniquely estimated K_m_ values, which were close to the measured values.

**Conclusions:**

MLAGO overcomes the major problems in parameter estimation, accelerates kinetic modeling, and thus ultimately leads to better understanding of complex cellular systems. The web application for our machine learning-based K_m_ predictor is accessible at https://sites.google.com/view/kazuhiro-maeda/software-tools-web-apps, which helps modelers perform MLAGO on their own parameter estimation tasks.

**Supplementary Information:**

The online version contains supplementary material available at 10.1186/s12859-022-05009-x.

## Background

Kinetic modeling is essential for understanding the dynamic behavior of biochemical networks [1]. Kinetic models consist of sets of ordinary differential equations (ODEs) with various kinetic parameters, such as Michaelis constants (K_m_s). K_m_ is the substrate concentration at which an enzyme operates at its half-maximal catalytic rate [2]. Most kinetic parameters have not been measured because they are traditionally measured in laborious low-throughput assays. Moreover, as kinetic parameters are measured under different experimental settings and often in vitro [3], even if the measured values are available, fine-tuning is still required to develop a realistic kinetic model that captures in vivo cellular behavior [4]. Kinetic parameter estimation has been a significant bottleneck in kinetic modeling [5].

Global optimization algorithms are often used to estimate kinetic parameters. In global optimization, the values of kinetic parameters are optimized so that models best fit the experimental data. Although different algorithms and software tools have been developed (e.g., [6–12]), the global optimization approach is time-consuming due to the large number of model parameters, nonlinear dynamics, and multiple local optima [13]. The conventional approach often yields unrealistic parameter values (e.g., extremely small or large values) because it simply seeks a better fit to the experimental data. Moreover, it often leads to nonunique solutions because different parameter sets allow a kinetic model to fit experimental data equally well [14, 15]. The problem of parameter non-identifiability makes the subsequent simulation studies difficult.

A few recent studies proposed alternative approaches: machine learning-based predictors for kinetic parameters. Heckmann et al. [16, 17] and Li et al. [18] employed machine and deep learning models to predict enzyme turnover numbers (k_cat_s). Kroll et al. [19] developed machine and deep learning models that predict K_m_ values. However, a few critical problems remain to be addressed. First, these predictors rely on a number of different features for substrates and enzymes, which are typically hard to obtain. For instance, the machine learning predictors proposed by [16, 17] require enzyme’s structural information, which is not broadly available for most enzymes. Moreover, the existing studies have not tested whether the predicted kinetic parameters are useful for kinetic modeling.

To overcome these limitations, we propose the Machine Learning-Aided Global Optimization (MLAGO) for K_m_ estimation of kinetic modeling. First, we develop a machine learning model for K_m_ prediction. Unlike the previous study [19], our machine learning model is based merely on EC number, KEGG Compound ID, and Organism ID. For the independent test dataset, there was only a four-fold difference between the measured and predicted K_m_ values on average. Then, we used the predicted K_m_ values as the reference values for the constrained global optimization-based parameter estimation. Through the real-world parameter estimation problems, we demonstrate that the MLAGO method can estimate K_m_ values with less computational cost than the conventional method. Moreover, we show that the MLAGO method could uniquely estimate realistic K_m_ values, which enable the kinetic models to fit experimental data.

## Results

### Kinetic modeling

Kinetic models for biochemical networks are formulated as ODEs:

1 $$\frac{{d{\mathbf{x}}}}{dt} = f(t,{\mathbf{x}},{\mathbf{p}}),$$

where *t* is time, **x** is a variable vector representing molecular concentrations, and **p** is a kinetic parameter vector including K_m_s. Parameter estimation is a task to find **p** that enables the model to fit the experimental data.

A model’s badness-of-fit (BOF) to the experimental data can be calculated as follows:

2 $$BOF({\mathbf{p}}) = \sqrt {n_{exp}^{ - 1} \cdot n_{point}^{ - 1} \cdot n_{mol}^{ - 1} \cdot \sum\limits_{i = 1}^{{n_{exp} }} {\sum\limits_{j = 1}^{{n_{point} }} {\sum\limits_{k = 1}^{{n_{mol} }} {\left( {\frac{{x_{i,j,k}^{sim} ({\mathbf{p}}) - x_{i,j,k}^{exp} }}{{x_{i,j,k}^{exp} }}} \right)^{2} } } } } ,$$

where **p** = (*p*_1_, *p*_2_, …) is a set of kinetic parameters used in the model. $x_{i,j,k}^{sim}$ and $x_{i,j,k}^{exp}$ are simulated and measured molecular concentrations, respectively. *n*_*exp*_, *n*_*point*_, and *n*_*mol*_ are the numbers of experimental conditions, data points, and measured molecular components, respectively.

In kinetic modeling, kinetic parameter values not only need to provide a good model fit to experimental data but also need to be biologically reasonable. If the models require unrealistic parameter values for a good fit, they fail to comply with reality. The implausibility of a set of estimated parameter values can be calculated as the root mean squared error (RMSE):

3 $$RMSE({\mathbf{q}},{\mathbf{q}}*) = \sqrt {n_{param}^{ - 1} \cdot \sum\limits_{i = 1}^{{n_{param} }} {\left( {q_{i} - q_{i} *} \right)^{2} } } .$$

As kinetic parameters take a large order of magnitude, we calculate RMSE on log_10_-scale. ${\mathbf{q}} = (q_{1} ,q_{2} , \ldots )$ and ${\mathbf{q}}* = (q_{1} *,q_{2} *, \ldots )$ are the log_10_-scaled estimated and reference parameter vectors, respectively. In other words, $q_{i} = \log_{10} (p_{i} )$ and $q_{i} * = \log_{10} (p_{i} *)$, where $p_{i}$ and $p_{i} *$ are the estimated and reference values, respectively. *Reference* refers to the values considered reasonable, such as measured values, and machine learning-predicted values.

Taken together, it is essential in kinetic modeling to find realistic kinetic parameter values that provide a good fit to experimental data, i.e., a kinetic parameter vector (**p**) that provides small BOF and RMSE values.

### Conventional global optimization approach

In the conventional global optimization approach, the parameter estimation problem is formulated as the following optimization problem:

4a $${\text{Minimize}}\quad \, BOF({\mathbf{p}}),$$

4b $${\text{Subject}}\;{\text{to}}\quad {\mathbf{p}}^{L} \le {\mathbf{p}} \le {\mathbf{p}}^{U} ,$$

where **p** = (*p*_1_, *p*_2_, …) is a set of kinetic parameters to be searched. BOF is the badness-of-fit [Eq. (2)]. **p**^*L*^ and **p**^*U*^ are the lower and upper bound vectors, respectively. Equation (4b) defines the search space. To cover the vast majority of K_m_ values (> 99%), we set a relatively large search space throughout this study: **p**^*L*^ = 10^−5^ mM and **p**^*U*^ = 10^3^ mM. The conventional approach simply seeks the parameter set that minimizes BOF, and the parameters can take any value within the lower and upper bounds without any penalties. Therefore, the estimated parameter values can be biologically unreasonable.

Global optimization algorithms are used for the global optimization approach, such as differential evolution [20], particle swarm optimization [21, 22], and scatter search [23, 24]. In this study, we used a particular real-coded genetic algorithm (RCGA), named the real-coded ensemble crossover star with just generation gap (REX^star^/JGG) [25]. REX^star^/JGG has been demonstrated competitive in parameter estimation tasks [6, 7, 26–28]. We employed the implementation provided by RCGAToolbox [7].

### Machine learning-aided global optimization (MLAGO)

In the conventional approach, the estimated parameter values can be biologically unrealistic (a large RMSE). In contrast, machine learning models may be able to predict reasonable parameter values based on available data on databases. However, the predicted values may not provide a good model fitting because the machine learning predictors do not take BOF into account.

**Fig. 1:**
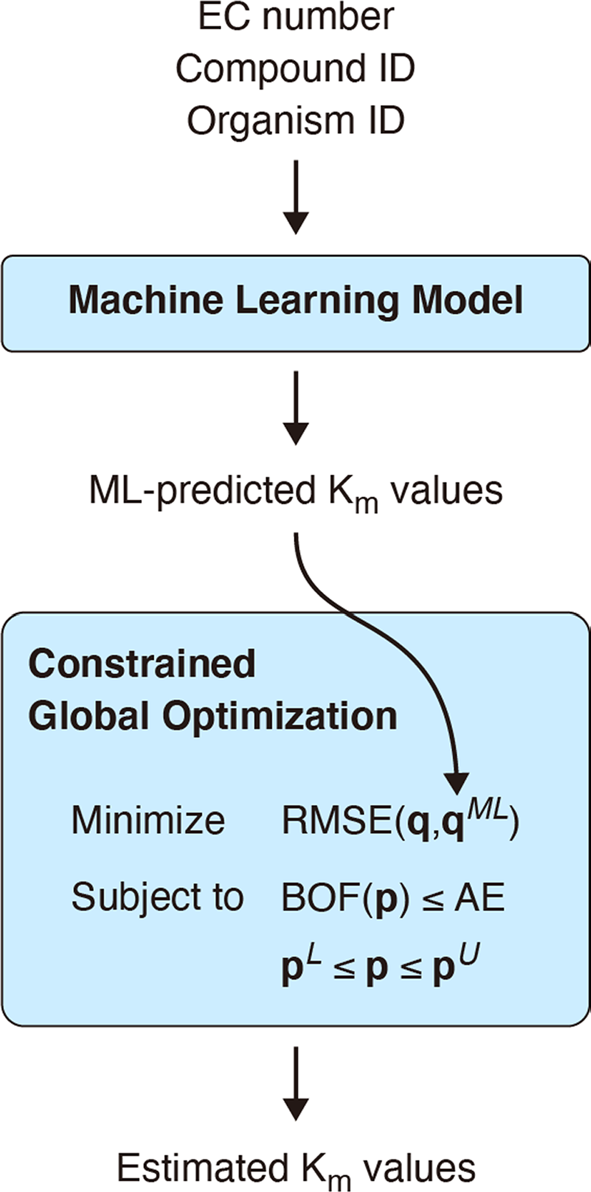
Overview of MLAGO, the machine learning-aided global optimization. Based on the enzyme information of EC number, Compound ID, and Organism ID, the machine learning model, which is trained and tested with 17,151 enzyme reaction data, predicts the values for unknown K_m_s in a kinetic model. The predicted K_m_ values are used as the reference values in the constrained global optimization. Finally, a global optimization algorithm estimates K_m_ values so that the kinetic model fits experimental data. **p** is a set of K_m_s to be searched, and **q** is log_10_-transformed **p**. **q**^*ML*^ is log_10_-transformed machine learning-predicted K_m_s. **p**^*L*^ and **p**^*U*^ are the lower and upper bound vectors, respectively. Abbreviations: ML (machine learning), RMSE (root mean squared error), and BOF (badness of fit), AE (allowable error). For RMSE, BOF, and AE, see the main text

To overcome these limitations, we propose the machine learning-aided global optimization (MLAGO) (Fig. 1). In this study, we focus on a specific type of kinetic parameters, K_m_s, which commonly appear in kinetic models. First, we use a machine learning model to predict unknown K_m_s in a kinetic model of interest. Then, we use the predicted K_m_s as reference values in the global optimization-based parameter estimation. More specifically, we formulate the parameter estimation task as the constrained global optimization problem:

5a $${\text{Minimize}}\;RMSE({\mathbf{q}},{\mathbf{q}}^{ML} ),$$

5b $${\text{Subject}}\;{\text{to}}\;BOF({\mathbf{p}}) \le AE,$$

5c $${\mathbf{p}}^{L} \le {\mathbf{p}} \le {\mathbf{p}}^{U} ,$$

where **p** = (*p*_1_, *p*_2_, …) is a set of kinetic parameters (i.e., K_m_s) to be searched, and **p**^*ML*^ = (*p*_1_^*ML*^, *p*_2_^*ML*^, …) is a set of the machine learning-predicted parameter values. ${\mathbf{q}} = (q_{1} ,q_{2} , \ldots )$ and ${\mathbf{q}}^{ML} = (q_{1}^{ML} ,q_{2}^{ML} , \ldots )$ are the log_10_-scaled **p** and **p**^*ML*^, respectively. RMSE and BOF are the root mean square error [Eq. (3)] and the badness-of-fit [Eq. (2)], respectively. AE is the allowable error (0.02 in this study). **p**^*L*^ and **p**^*U*^ are the lower and upper bound vectors, respectively. To cover the vast majority of K_m_ values, we use a relatively large search space, e.g., **p**^*L*^ = 10^–5^ mM and **p**^*U*^ = 10^3^ mM. In this study, we call the parameter sets that satisfy Eq. (5b) and (5c) as “solution parameter sets” or “solutions.” The constrained global optimization aims to minimize RMSE between K_m_s to be searched and machine learning-predicted K_m_ values while keeping a sufficiently good model fit to experimental data. Minimization of RMSE works as “regularization” [15], which helps the global optimization algorithm to estimate a unique solution. Whether the MLAGO works well or not depends on how accurate the machine learning predictor can predict K_m_ values for **p**^*ML*^.

### Developing the machine learning-based K_m_ predictor

#### Data preparation

To develop machine learning-based K_m_ predictors, we employed the well-curated K_m_ dataset provided by Bar-Even et al. [29]. The dataset is publicly available as a Supporting Information of [29]. Briefly, this dataset was originally obtained from BRENDA [30, 31] and contained 31,162 K_m_ values for different combinations of EC numbers, substrates, organisms, and experimental conditions (e.g., temperature and pH). The entries for mutated enzymes and non-natural substrates had already been removed. KEGG Compound IDs and Organism IDs had been assigned to substrates and organisms, respectively [32]. We did not use temperature or pH for our machine learning models because they do not contribute to prediction performance (see Discussion). Next, we merged duplicated entries (i.e., entries with identical EC numbers, Compound IDs, and Organism IDs) into a single entry. We took the geometric mean for K_m_ values across duplicated entries. Then, we removed 17 entries related to K_m_s for the two kinetic models employed in the benchmark experiments. As a result, we obtained K_m_ dataset with 17,151 entries (2,588 unique EC numbers, 1,612 unique Compound IDs, and 2212 unique Organism IDs). We randomly divided the dataset into the training and test datasets with a ratio of 4:1.

#### Feature encoding

**Fig. 2:**
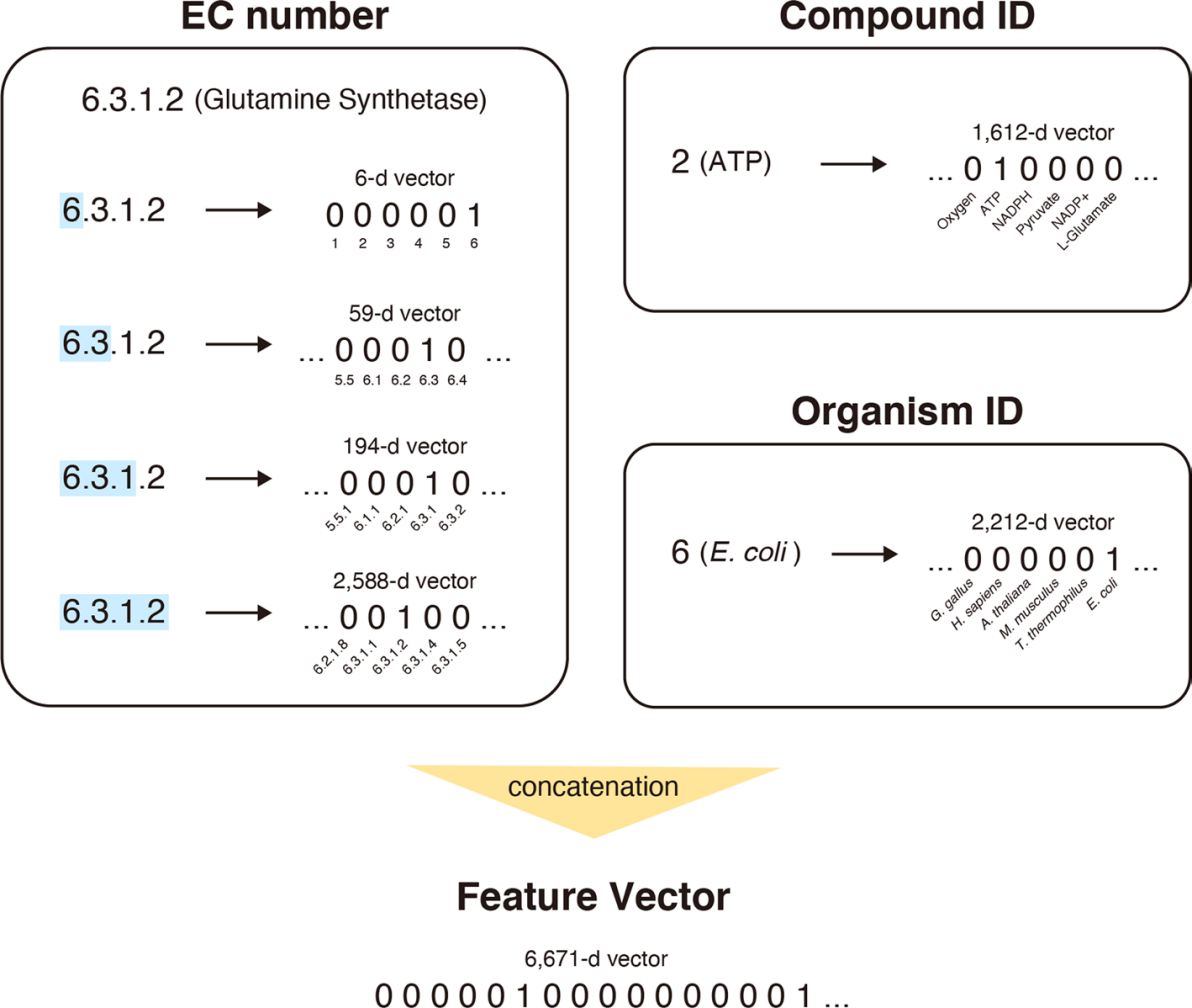
Feature encoding. Glutamine synthetase (EC 6.3.1.2), ATP, and *E. coli* were shown for illustrative purpose

For feature encoding, we took the most straightforward approach: the one-hot encoding (Fig. 2). As our dataset contained 1612 different compounds, we used a 1612-dimensional binary vector to encode a compound. In this vector, only an element corresponding to a particular Compound ID is one, and the remaining elements are zeros. In the same way, we employed a 2212-dimensional binary vector for Organism ID.

To retain the hierarchical information, we took a slightly different approach to encode EC numbers. We used four binary vectors to encode an EC number: The vectors for the first digit, the first two digits, the first three digits, and the entire four digits. As our dataset contained six different first digits (i.e., EC 1 to 6), we used a 6-dimensional vector to encode the first digit. As our data contained 59 different first two digits (e.g., EC 1.1, 1.2, 3.1, and 6.3), we used a 59-dimensional vector to encode the first two digits. Similarly, we used 194-dimensional and 2588-dimensional vectors for the first three digits and the entire four digits, respectively. Then, to represent an EC number, we concatenated these four vectors, i.e., the vectors for the first digit, the first two digits, the first three digits, and the entire four digits.

As a result of the hierarchical encoding, the feature vector for EC 1.1.1.1 is more similar to that for EC 1.1.1.2 than that for EC 2.1.1.1: The feature vector for EC 1.1.1.1 is generated by substituting 1 for the elements corresponding to “1”, “1.1”, “1.1.1”, and “1.1.1.1”. Namely, the 1st, 7th (6 + 1), 66th (6 + 59 + 1), and 260th (6 + 59 + 194 + 1) elements are one. The remaining elements are zero. Similarly, the feature vector for EC 1.1.1.2 is generated by substituting 1 for the elements corresponding to “1”, “1.1”, “1.1.1”, and “1.1.1.2”, i.e., the 1^st^, 7th (6 + 1), 66th (6 + 59 + 1), and 261st (6 + 59 + 194 + 2) elements. The feature vector for EC 2.1.1.1 is generated by entering 1 to the elements corresponding to “2”, “2.1”, “2.1.1”, and “2.1.1.1”, i.e., the 2nd, 28th (6 + 22), 162nd (6 + 59 + 97), and 1136th (6 + 59 + 194 + 877) elements. Thus, the vectors for EC 1.1.1.1 and EC 1.1.1.2 have 1 for the three common elements (i.e., the 1st, 7th, and 66th elements). Meanwhile, the vectors for EC 1.1.1.1 and EC 2.1.1.1 do not have 1 for any common elements.

Taken together, an entry from our dataset has a 6671-dimensional binary feature vector in total. The task that machine learning models perform in this study is to predict K_m_ values based on the 6671-dimensional binary feature vectors.

### Evaluation of machine learning-based K_m_ predictors

We employed five machine learning algorithms: *k*-nearest neighbors algorithm, elastic net, random forest model, gradient boosting model, and TabNet (see Methods). First, we performed hyperparameter tuning for the five models through five-fold cross-validation on the training dataset. Next, we trained the machine learning models with the best hyperparameter settings and all the training data. Finally, we tested the predictive performance on the test dataset.

**Fig. 3:**
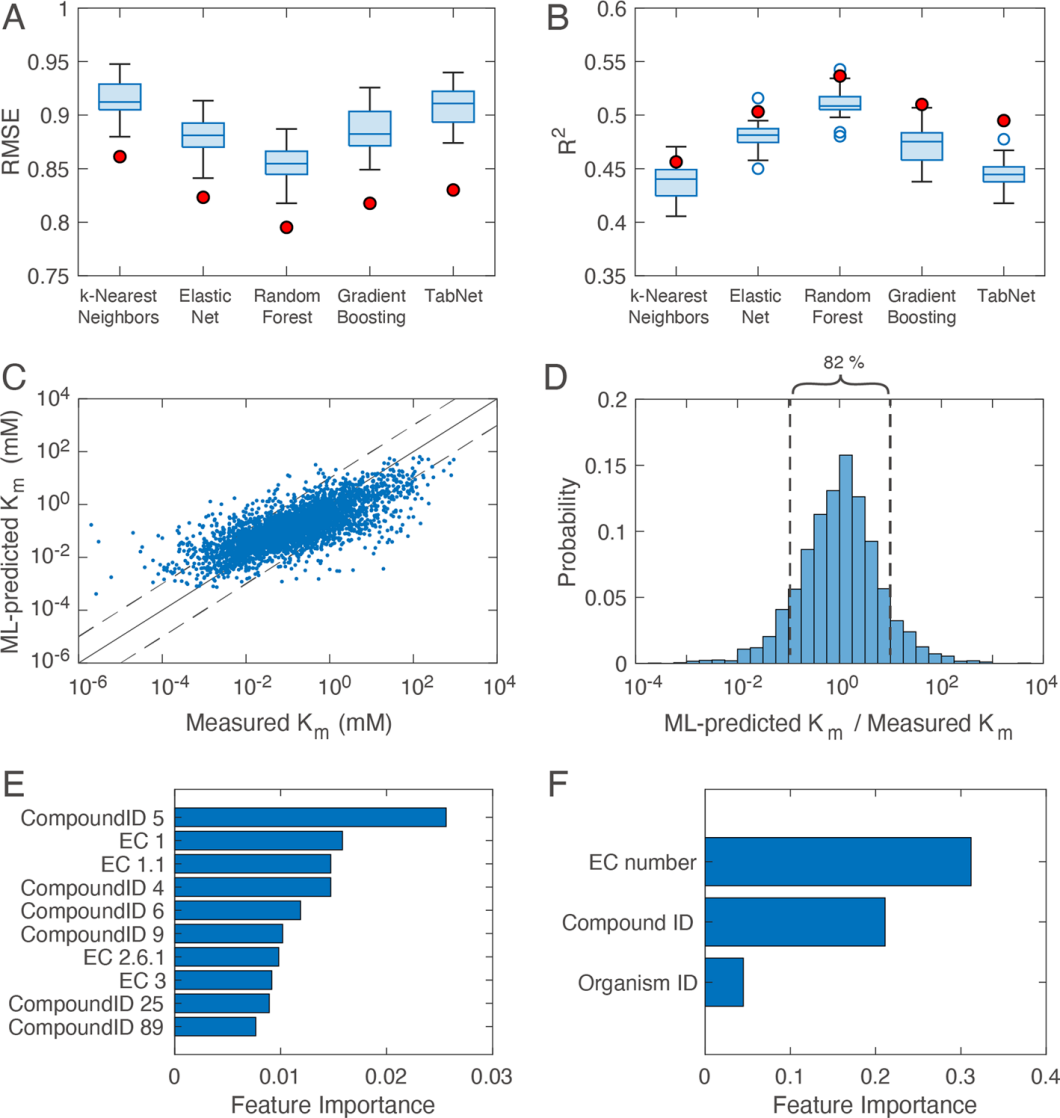
Performance of the machine learning-based K_m_ predictors. **A** RMSE. **B** R^2^. The values in (**A**) and (**B**) were calculated with the best hyperparameter setting for each model. The boxes, whiskers, and open circles are for four independent rounds of five-fold cross-validation with the training dataset. The red circles are for the test dataset, which was not used for hyperparameter tuning. **C** Scatter plot of K_m_ values of the test dataset predicted with the random forest model versus the experimental values. **D** Histogram of the ratio of the predicted K_m_ values to the experimental values. **E** Top 10-ranked important features in the random forest model. **F** Feature importance for each feature class. Feature importance in (**E**) and (**F**) was calculated by the permutation-based method

The best hyperparameter settings are summarized in Additional file 1: Table S1, and model performance is shown in Fig. 3. The random forest model achieved the best performance in the cross-validation and the independent test (Fig. 3A, B). In the independent test with the test dataset, the random forest model achieved RMSE = 0.795 and R^2^ = 0.536. Since the random forest model achieved the best performance, we used it for further analyses.

Figure 3C is the scatter plot of K_m_ values of the test datasets predicted with the random forest model versus measured K_m_ values. The predicted and measured values were different by four-fold on average on either side of the measured values. The deviations in 82% of K_m_s were less than ten-fold on either side of the measured values (Fig. 3D). Next, we investigated important features for prediction. As shown in Fig. 3E, all the top 10-ranked features were the features related to EC numbers or Compound IDs. Indeed, the sum of the feature importance values for the Organism ID-derived features is much smaller than those for EC number and Compound ID-derived features (Fig. 3F), indicating that the K_m_ predictor mainly uses EC number and Compound ID.

In summary, we developed machine learning models for K_m_ prediction, which relies merely on EC number, Compound ID, and Organism ID. The random forest model achieved the best prediction scores.

### Machine learning-predicted K_m_ values do not provide a good model fit

**Fig. 4:**
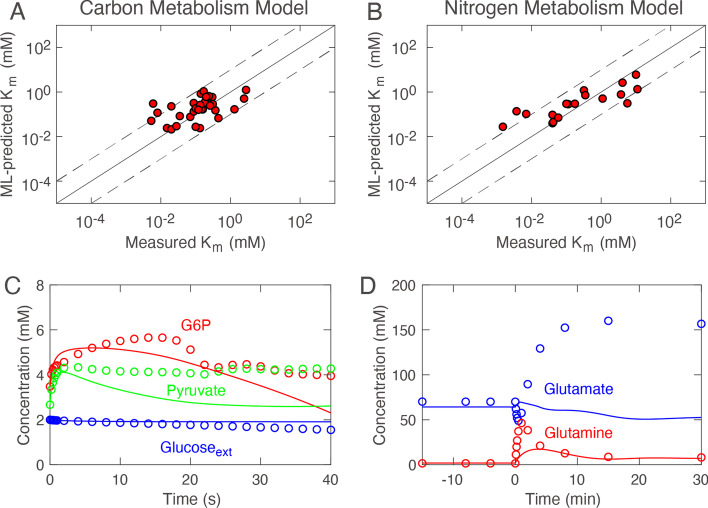
Machine learning prediction of K_m_ for the benchmark models. **A** and **B** Scatter plots of predicted K_m_ values versus the experimental values for the carbon and nitrogen metabolism models. **C** and **D** Simulation results of the carbon and nitrogen metabolism models with the machine learning-predicted K_m_ values. The circles and lines represent experimental data and simulation, respectively. Only important molecular components are shown for clarity

Next, we investigated whether the machine learning-predicted K_m_ values as they are can be used for kinetic modeling. We considered the two real-world kinetic models: The carbon metabolism model [33] and the nitrogen metabolism model [26] (see Methods). Using the machine learning K_m_ predictor, we estimated 32 and 18 K_m_s in the carbon and nitrogen metabolism models, respectively. Please note that these K_m_s were not included in the training or test datasets. However, the machine learning model predicted those K_m_ values with good accuracy (Fig. 4A, B): RMSE = 0.616 and 0.727 for the carbon and nitrogen metabolism models, respectively. Encouraged by this result, we tested whether the kinetic models with the predicted K_m_ values reproduce the experimentally observed behaviors. As shown in Fig. 4C, D, the kinetic models did not fit the experimental data (BOF > 1.143). Therefore, we concluded that machine learning-predicted K_m_ values could not be used for kinetic models as they were.

### MLAGO outperforms the conventional global optimization approach

Next, we investigated whether the machine learning-predicted K_m_ values can be used as the reference values for MLAGO. We compared the MLAGO approach and the conventional global optimization approach. Again, we employed the carbon and nitrogen metabolism models as benchmark problems. We used the machine learning predictor (the random forest model) to predict K_m_ values and used them as the reference values for the MLAGO approach [**p**^*ML*^ in Eq. (5)]. We employed REX^star^/JGG as a global optimization algorithm both for the MLAGO and conventional approaches. Each approach was carried out ten times for each kinetic model.

**Fig. 5:**
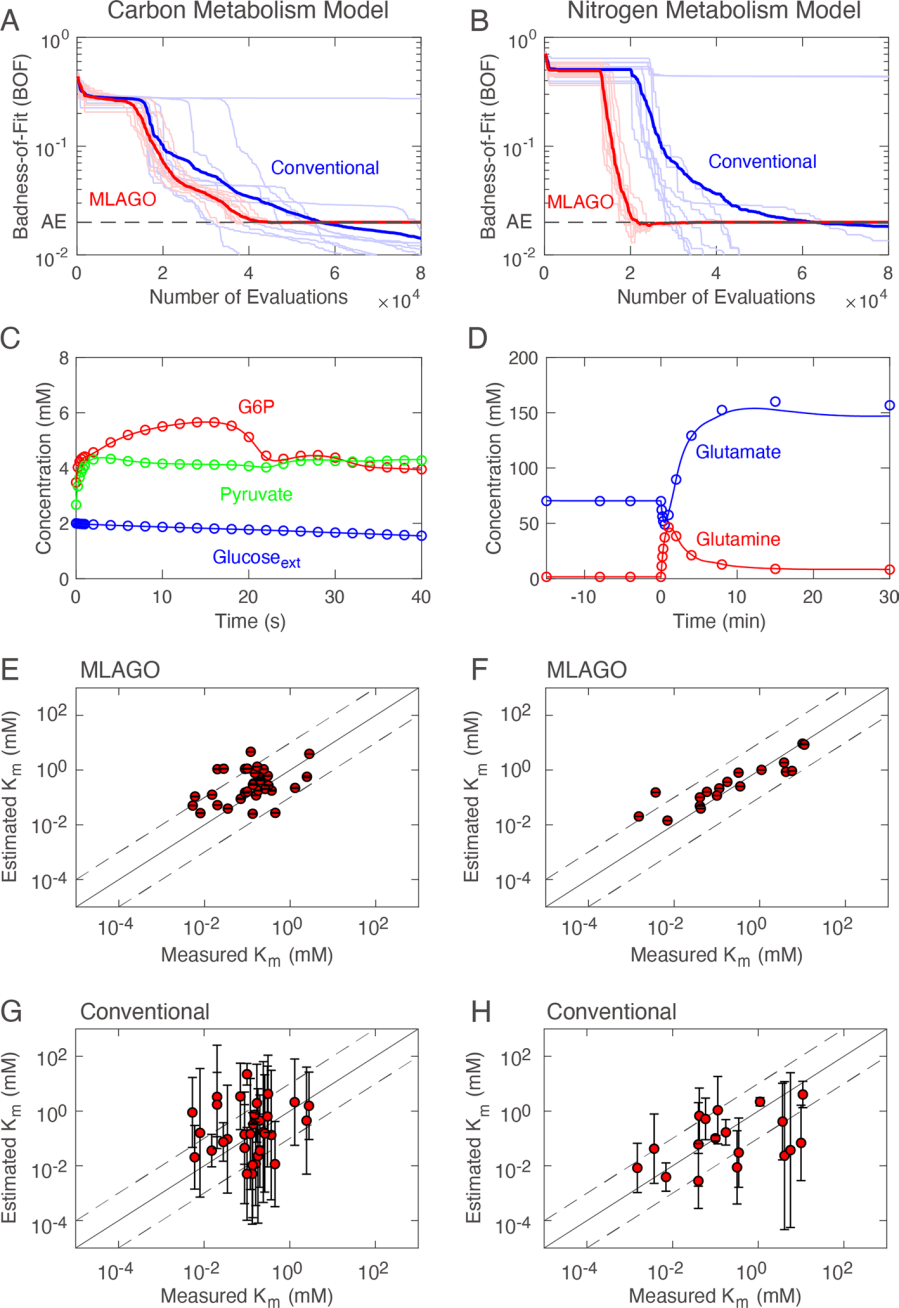
Performance of the MLAGO method. **A** and **B** Convergence curves for the MLAGO method (red) and conventional method (blue). The thin lines with light colors represent independent trials, and the thick lines with strong colors represent the geometric mean of these trials. The dashed black lines represent the allowable error (AE). The number of evaluations indicates the number of simulations performed during the global optimization. **C** and **D** Simulation results of the carbon and nitrogen metabolism models with the K_m_ values estimated by the MLAGO method. The circles and lines represent experimental data and simulation, respectively. Only important molecular components are shown for clarity. **E** and **F** Scatter plots of K_m_ values estimated by the MLAGO method. **G** and **H** Scatter plots of K_m_ values estimated by the conventional method. In (**E**)–(**H**), the circles represent mean values, and error bars represent ± standard deviation (*n* = 10). In (**E**) and (**F**), the error bars are not clearly visible because the standard deviation is small

The convergence curves are shown in Fig. 5A, B. The MLAGO method found solutions (BOF ≤ AE) with less computational costs than the conventional method. Please note that a set of K_m_ values with BOF = AE already provides a sufficiently good fit to experimental data. Once BOF ≤ AE is achieved, the MLAGO approach focuses on reducing RMSE, and thus BOF stays slightly below or equal to AE. The MLAGO approach found solutions for all ten trials for both the carbon and nitrogen metabolism models. In contrast, the conventional method failed to find solutions in one trial for the carbon metabolism model and three trials for the nitrogen metabolism model. We confirmed that the K_m_ values estimated by the MLAGO (Fig. 5C, D) and conventional methods (not shown) could provide a good model fit to experimental data.

Next, we checked whether the estimated K_m_ values were close to measured values. The MLAGO method obtained K_m_ values close to their measured values (Fig. 5E, F). Indeed, the RMSE values between the estimated and measured values were small, and the obtained parameter sets were almost identical for all the ten trials: RMSE = 0.787 ± 0.002 and RMSE = 0.571 ± 0.001 for the carbon and nitrogen metabolism models, respectively (*n* = 10; ± SD). In contrast, the K_m_ values estimated by the conventional method were very different from their measured values, and the estimated values varied depending on the trials (Fig. 5G, H): RMSE = 1.879 ± 0.196 for the carbon metabolism model and RMSE = 1.698 ± 0.283 for the nitrogen metabolism model. Most K_m_ values estimated by the MLAGO were within a reasonable range: For the carbon metabolism model, the deviations in 78% of the K_m_s were less than ten-fold on either side of the measured values. For the nitrogen metabolism model, it was 89%.

In summary, the MLAGO approach estimated K_m_ values with less computational cost than the conventional approach. Moreover, the MLAGO approach almost uniquely identified K_m_ values, most of which were close to the measured values.

## Discussion

We proposed a hybrid parameter estimation technique based on machine learning and global optimization, named MLAGO. In kinetic modeling, the global optimization approach has been used for parameter estimation. However, the conventional approach had three major problems: (i) It is computationally costly. (ii) It often yields unrealistic parameter values. (iii) It has difficulty identifying a unique solution. To solve these problems, we proposed the MLAGO approach integrating global optimization and machine learning. The main idea is to use the machine learning-predicted K_m_ values as the reference values for constrained global optimization. The MLAGO approach updates K_m_ values to improve model fit to experimental data while keeping them close to the values predicted by machine learning. To implement the MLAGO approach, we developed a machine learning model for K_m_ prediction using three factors: EC number, Compound ID, and Organism ID. Through real-world benchmark problems, we confirmed that the MLAGO approach was superior to the conventional approach: The MLAGO approach found a solution with less computational cost than the conventional approach, and the solution was close to measured K_m_ values. Moreover, the MLAGO approach estimated almost the identical solution for all the independent trials. To our knowledge, this work is the first study to integrate global optimization and machine learning for kinetic parameter estimation. Machine learning-predicted, realistic K_m_ values can be helpful for kinetic modeling because unrealistic K_m_ values may lead to wrong predictions.

As a further experiment, we investigated whether we could improve the machine learning K_m_ predictors by adding different features. Specifically, we added temperature, pH, amino acid sequence motifs (Pfam domain [34]), and pathway information (KEGG Pathway ID [32]). Temperature and pathway information slightly improved the prediction score; however, the improvement was not statistically significant (*p* > 0.05). Therefore, the prediction scores achieved by our best model (RMSE = 0.795 and R^2^ = 0.536) may be close to the best possible prediction scores, considering the number and quality of datasets available in public databases. This speculation is supported by the fact that Kroll et al. took a very different approach and achieved performance scores comparable to ours: MSE = 0.65 (i.e., RMSE = 0.81) and R^2^ = 0.53 [19].

As another experiment, we investigated whether the carbon and nitrogen metabolism models with measured K_m_ values reproduce their experimentally observed behaviors. As shown in Additional file 1: Fig S1, they failed to do so (BOF > 0.509). One reason for the misfit is that K_m_ values are usually measured under enzyme-specific in vitro conditions. Thus, K_m_ values need to be tuned by global optimization for better model fit. Indeed, the measured K_m_ values in our datasets are different from the “original” K_m_ values given in the carbon and nitrogen metabolism models. The RMSE between the original and measured values was 0.857 for the carbon metabolism model and 0.111 for the nitrogen metabolism model. For the carbon metabolism model, the RMSE between the machine learning-predicted K_m_ values and the measured values was 0.616. Therefore, K_m_ values for the carbon metabolism model were greatly improved by the machine learning K_m_ predictor.

Not only K_m_s but also k_cat_s and V_max_es are often estimated in parameter estimation. We conducted additional computational experiments to investigate whether MLAGO can uniquely estimate K_m_ values even along with k_cat_s and V_max_es. k_cat_ and V_max_ values were searched in global optimization but not considered in the RMSE calculation [Eq. (5a)] because measured values are rarely available for them. As shown in Additional file 1: Fig S2, MLAGO estimated K_m_ values almost uniquely even if k_cat_s and V_max_es are searched: RMSE = 0.650 ± 0.018 and RMSE = 0.596 ± 0.001 for the carbon and nitrogen metabolism models, respectively (*n* = 10; ± SD).

It is difficult to compare the prediction quality of our K_m_ predictor with Kroll’s [19] as their and our datasets are not exactly the same due to differences in employed features. Nevertheless, it is notable that our K_m_ predictor achieved a good prediction score, RMSE = 0.795, compared to RMSE = 0.81 by Kroll et al. In their article, Kroll et al. provided genome-scale K_m_ predictions for 47 model organisms. Thus, we investigated whether their predicted K_m_ values could be used for the carbon and nitrogen metabolism models. Specifically, we used the predicted K_m_ values provided for an *E. coli* genome-scale metabolic model (iAF1260). We found that the RMSE between their prediction and the measured values are relatively large: RMSE = 0.961 for the carbon metabolism model and RMSE = 1.328 for the nitrogen metabolism model. As mentioned above, our K_m_ predictor achieved better scores: RMSE = 0.616 for the carbon metabolism model and RMSE = 0.727 for the nitrogen metabolism model.

Kroll et al. [19] and we took a different approach to K_m_ prediction. Kroll et al. combined deep and machine learning models. They employed deep learning for feature encoding: a task-specific molecular fingerprint of the substrate and deep numerical representation of the enzyme’s amino acid sequence [19]. In their approach, substrate’s structure and enzyme’s amino acid sequence were converted into a 52-dimensional fingerprint vector and a 1,900-dimensional UniRep [35] vector, respectively. Then, the resultant 1,952-dimensional feature vector was used by the gradient boosting model (XGBoost). In contrast, we employed simple feature encoding and machine learning methods, i.e., the one-hot encoding and random forest. In our approach, EC number, Compound ID, and Organism ID were converted into 2847-dimensional, 1612-dimensional, and 2212-dimensional binary vectors, respectively. The resultant 6671-dimensional feature vector was used for the random forest. It may be surprising that our simple machine learning model achieved a good performance. We encoded EC number so that the feature vector retains the information on enzyme classification. We think this encoding method contributed to the prediction performance. Indeed, the feature importance for EC number is larger than that for Compound ID and Organism ID in our machine-learning K_m_ predictor (Fig. 3F), which is in contrast to Kroll’s K_m_ predictor in which the substrate information is more important than enzyme information.

The advantage of our K_m_ predictor over Kroll’s [19] is that ours does not require compound’s structural information or enzyme’s amino acid sequence. Our predictor requires only EC number, Compound ID, and Organism ID, which are easily available for kinetic modelers. Nonetheless, our predictor has a limitation: although the dataset used in this study covers a vast number of enzymes, substrates, and organisms (2588 EC numbers, 1612 Compound IDs, and 2212 Organism IDs), our K_m_ predictor would probably show poor performance on uncommon enzymes, substrates, and organisms that were not included in the training data. Moreover, EC numbers have not been assigned to newly found enzymes. Similarly, Compound IDs and Organism IDs may not be assigned to rare substrates and organisms. Our K_m_ predictor cannot handle these enzymes, compounds, and organisms without EC number, Compound ID, and Organism ID. Therefore, our approach is not applicable to rare enzymes and compounds. In contrast, Kroll’s approach is organism-independent and applicable as long as compound’s structure and enzyme’s amino acid sequence are available.

We successfully predicted K_m_ values without chemical, physicochemical, or structural information. This fact implies that enzymes with similar EC numbers (i.e., enzymes that catalyze similar reactions) tend to have similar K_m_ values. Also, which substrate is involved is an essential factor to determine K_m_ values. Indeed, K_m_ values and physiological substrate concentrations may have co-evolved to match each other [29, 36].

Generally speaking, the gradient boosting model and TabNet tend to outperform the random forest model. In this study, we tested 864 and 172 hyperparameter combinations for the gradient boosting model and TabNet, respectively. However, despite the intensive hyperparameter tuning, we could not find any hyperparameter settings for these models to outcompete the random forest model. This may be due to the limited size of the training dataset (13,721 entries) compared to the dimension of the feature vector (6,671). In general, more complex models need more data.

There are two limitations in the MLAGO approach. First, our machine learning model is relatively poor at predicting extremely small or large K_m_ values. The K_m_ predictor tends to predict a slightly higher value for the K_m_s whose measured values are less than 0.01 mM, and a slightly lower value for the K_m_s whose measured values are more than 1 mM (Fig. 3C and Fig. 4A, B). Second, the goal of parameter estimation is to simultaneously achieve accurate K_m_ estimation and model fitting, but it is not always achievable. Indeed, the accuracy of K_m_ estimation and quality of model fitting are trade-off in some cases, including the carbon metabolism model (Additional file 1: Fig S3A). The trade-off is caused by different reasons, such as inaccurate experimental data or flaws in kinetic models. In the trade-off cases, AE needs to be tuned to balance the accuracy of K_m_ estimation and model fitting. Modelers can also use the trade-off as an indicator of inconsistency between a kinetic model and experimental data.

## Conclusions

The previous studies [16–18] demonstrated that deep learning-based k_cat_ prediction improved genome-scale constraint-based metabolic models. However, whether machine learning-based K_m_ prediction is helpful to kinetic modeling had not been tested. In this study, we showed that machine learning-predicted K_m_ values can serve as the reference values for the constrained optimization-based parameter estimation. We conclude that the MLAGO approach improves parameter estimation in kinetic modeling, leading to better understanding of complex cellular systems. The web application for the machine learning-based K_m_ predictor is accessible at https://sites.google.com/view/kazuhiro-maeda/software-tools-web-apps, which helps modelers perform MLAGO on their own parameter estimation tasks. The K_m_ predictor is applicable not only to kinetic modeling but also to diverse applications, including Enzymology and Bioindustry.

## Methods

### Machine learning algorithms

We employed five machine learning algorithms: *k*-nearest neighbors algorithm, elastic net, random forest model, gradient boosting model, and TabNet. The *k*-nearest neighbors algorithm is the simplest: the output is the average of the values of *k* nearest neighbors. The elastic net is a regularized regression method that linearly combines the L_1_ and L_2_ penalties. The random forest and gradient boosting are ensemble learning methods that operate by constructing a number of decision trees at training. TabNet is an interpretable canonical deep learning architecture for tabular data [37]. We used scikit-learn [38] for the *k*-nearest neighbors algorithm, elastic net, and random forest model. We employed XGBoost [39] for the gradient boosting model. For TabNet, we used an implementation provided in GitHub [40].

### Performance criteria

To evaluate the performance of machine learning models, we use the two measures: RMSE [Eq. (3)] and the coefficient of determination (R^2^):

6 $$R^{2} ({\mathbf{q}},{\mathbf{q}}*) = 1 - \frac{{\sum\nolimits_{i = 1}^{{n_{param} }} {\left( {q_{i} - q_{i} *} \right)^{2} } }}{{\sum\nolimits_{i = 1}^{{n_{param} }} {\left( {q_{i} - \overline{q*} } \right)^{2} } }},$$

where $\overline{q*} = n_{param}^{ - 1} \cdot \sum\nolimits_{i = 1}^{{n_{param} }} {q_{i} *}$. ${\mathbf{q}} = (q_{1} ,q_{2} , \ldots )$ and ${\mathbf{q}}* = (q_{1} *,q_{2} *, \ldots )$ are the log_10_-scaled estimated and experimentally measured K_m_ vectors, respectively.

### Benchmark problems

We employed two kinetic models for benchmarking the MLAGO approach presented in this study. The carbon metabolism model [33] contains the glycolysis and pentose-phosphate pathway and consists of 18 variables and 137 model parameters. The nitrogen metabolism model [26] contains the ammonium transport and glutamate and glutamine production pathways and consists of 13 variables and 111 kinetic parameters. The main features of the carbon metabolism model [33] and the nitrogen metabolism model [26] are summarized in Additional file 1: Table S2. We chose these models because (i) they are realistic models that can quantitatively reproduce changes in metabolite concentrations, (ii) their simulation models were available from the BioModels database [41], and (iii) their simulations are computationally feasible.

In the benchmark experiments, we estimated 32 K_m_s in the carbon metabolism model and 18 K_m_s in the nitrogen metabolism model (see Additional file 1: Table S2). We chose these K_m_s as targets because they have been measured, and thus we can check estimation accuracy. For simplicity, we generated quasi-experimental data [$x_{i,j,k}^{exp}$ in Eq. (2)] by performing simulations with the original K_m_ values given in the models.

## Supplementary Information

**Additional file 1**. Tables S1–S2 and Figs S1–S3.

## Data Availability

The datasets generated and/or analyzed during the current study are available in GitHub, https://github.com/kmaeda16/MLAGO-data
