# Supplementary material for: MLAGO: machine learning-aided global optimization for Michaelis constant estimation of kinetic modeling
Source: BMC Bioinformatics. 2022 Nov 1;23:455. doi: 10.1186/s12859-022-05009-x (PMC9624028; doi:10.1186/s12859-022-05009-x)
Supplement: Supplementary file 1 — Additional file 1. Tables S1–S2 and Figs S1–S3. [file 12859_2022_5009_MOESM1_ESM.pdf]

MLAGO: Machine learning-aided global optimization for Michaelis constant estimation of kinetic modeling

Kazuhiro Maeda, Aoi Hatae, Yukie Sakai, Fred C. Boogerd, and Hiroyuki Kurata

Additional file 1

Table S1. Best hyperparameter settings for machine learning models

Model Name	Hyperparameter Setting
k-Nearest Neighbors	n_neighbors = 5
Elastic Net	alpha = 1e-4, l1_ratio = 2e-1
Random Forest	n_estimators = 300, max_features = 0.01
Gradient Boosting	subsample = 1.0, colsample_bytree = 0.9, reg_alpha = 1e-3, reg_lambda = 1e+0, min_child_weight = 2, max_depth = 36, gamma = 0.5
TabNet	n_d = 12, n_a = 12, momentum = 0.02, n_steps = 3, batch_size = 1024, virtual_batch_size = 32

Table S2. Main features of benchmark models

Model Name	Carbon metabolism model	Nitrogen metabolism model
Total Parameters	137	111
Parameters to be estimated (Figure 5)	32 ($K_{PGI,g6p}$, $K_{PGI,f6p}$, $K_{PGM,g6p}$, $K_{PGM,g1p}$, $K_{G6PDH,g6p}$, $K_{G6PDH,nadp}$, $K_{PFK,atp,s}$, $K_{PFK,f6p,s}$, $K_{ALDO,fdp}$, $K_{ALDO,gap}$, $K_{ALDO,dhap}$, $K_{GAPDH,gap}$, $K_{GAPDH,nad}$, $K_{GAPDH,nadh}$, $K_{TIS,dhap}$, $K_{TIS,gap}$, $K_{PGK,adp}$, $K_{PGK,atp}$, $K_{PGK,pgp}$, $K_{PGK,3pg}$, $K_{PGluMu,3pg}$, $K_{PGluMu,2pg}$, $K_{ENO,2pg}$, $K_{ENO,pep}$, $K_{PK,pep}$, $K_{PK,adp}$, $K_{DAHPS,e4p}$, $K_{DAHPS,pep}$, $K_{PGDH,6pg}$, $K_{PGDH,nadp}$, $K_{G1PAT,atp}$, and $K_{G1PAT,g1p}$)	18 (K_{ututp}, K_{utippi}, K_{gdhog}, K_{gdhnh}, K_{gdhglu}, $K_{gdhnadph}$, $K_{gdhnadp}$, K_{goggln}, K_{gogog}, $K_{gognadph}$, K_{gogglu}, $K_{gognadp}$, K_{gsatp}, K_{gsglu}, K_{gsnh}, K_{gsadp}, K_{gspi}, and K_{gsgln})
Parameters to be estimated (Figure S2)	46 (K_ms shown above and $r_{max,PGI}$, $r_{max,PGM}$, $r_{max,G6PDH}$, $r_{max,PFK}$, $r_{max,ALDO}$, $r_{max,GAPDH}$, $r_{max,TIS}$, $r_{max,PGK}$, $r_{max,PGluMu}$, $r_{max,ENO}$, $r_{max,PK}$, $r_{max,DAHPS}$, $r_{max,PGDH}$, $r_{max,G1PAT}$)	23 (K_ms shown above and $k_{catutglnb}$, $k_{catutglnk}$, V_{gdh}, V_{gog}, k_{catgs})
Dynamic States	18	13
Observed States	18	2 (Glutamate and glutamine)
Experiments	1	3 (Wild Type, ΔGDH, and ΔGOGAT)
Data Points	468	84
Reference	Chassagnole et al., Biotechnol Bioeng, 2002	Maeda et al., NPJ Syst Biol Appl, 2019

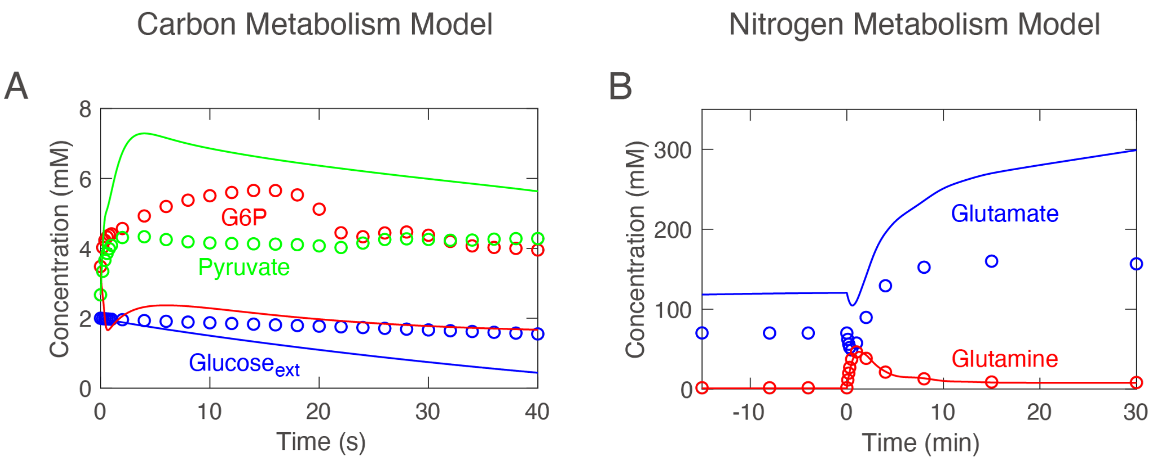

Figure S1. Simulations with the measured K_m values

Simulation results of (A) the carbon metabolism model and (B) nitrogen metabolism model, with the measured K_m values. The circles and lines represent experimental data and simulation, respectively. Only important molecular components are shown for clarity.

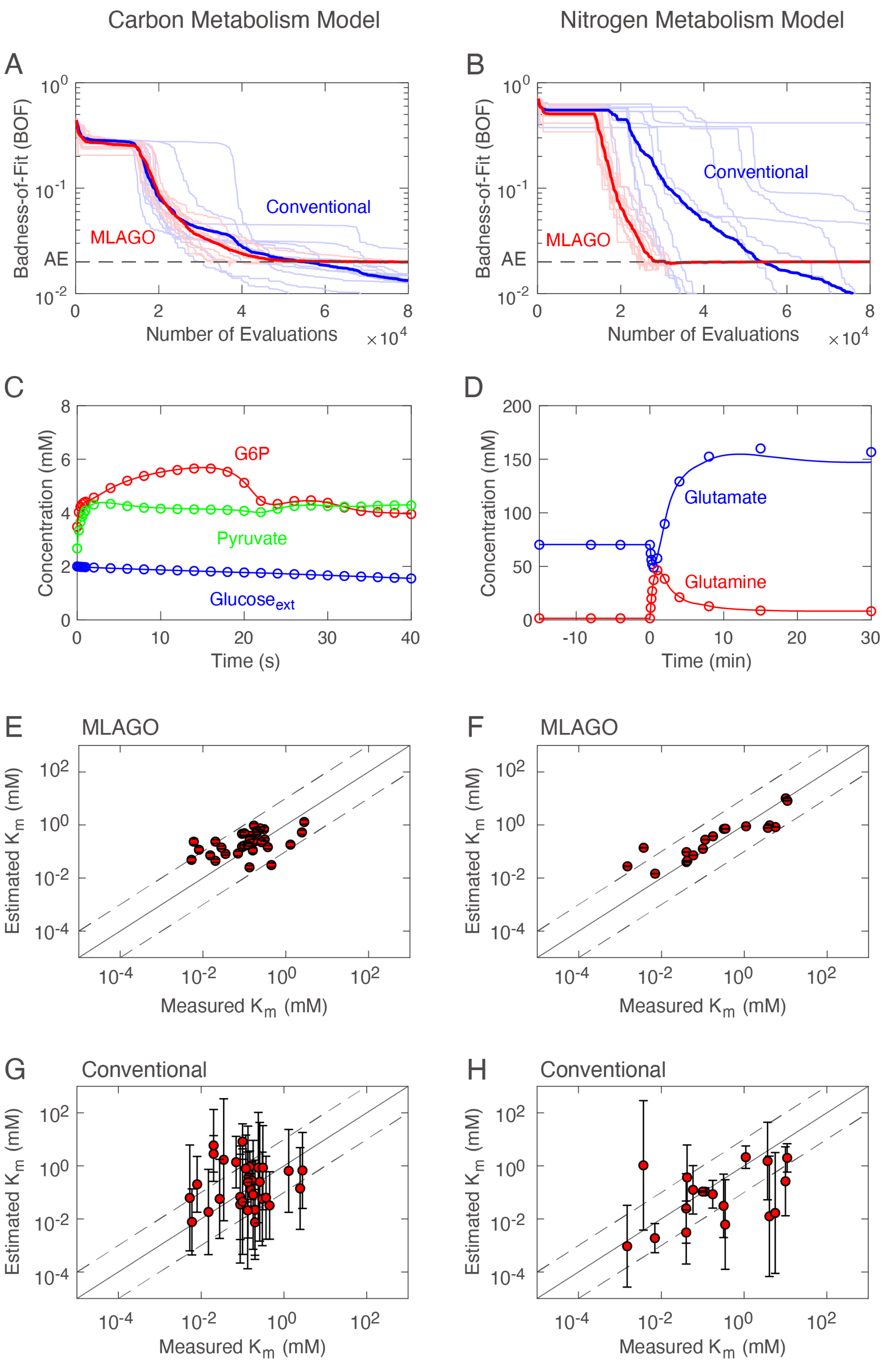
Carbon Metabolism Model
Nitrogen Metabolism Model
A
B
Badness-of-Fit (BOF)
Conventional
MLAGO
AE
Number of Evaluations
C
D
Concentration (mM)
G6P
Pyruvate
Glucose_ext
Glutamate
Glutamine
Time (s)
Time (min)
E
F
MLAGO
Estimated K_m (mM)
Measured K_m (mM)
G
H
Conventional

Figure S2. K_m values were estimated along with k_{cat}s and V_{max}es

(A) and (B): Convergence curves for the MLAGO method (red) and conventional method (blue). The thin lines with light colors represent independent trials, and the thick lines with strong colors represent the geometric mean of these trials. The dashed black lines represent the allowable error (AE). The number of evaluations indicates the number of simulations performed during the global optimization. (C) and (D): Simulation results of the carbon and nitrogen metabolism models with the K_m values estimated by the MLAGO method. The circles and lines represent experimental data and simulation, respectively. Only important molecular components are shown for clarity. (E) and (F): Scatter plot of K_m values estimated by the MLAGO method. (G) and (H): Scatter plot of K_m values estimated by the conventional method. In (E) – (H), the circles represent mean values, and error bars represent ± standard deviation (n = 10). In (E) and (F), the error bars are not clearly visible because the standard deviation is small.

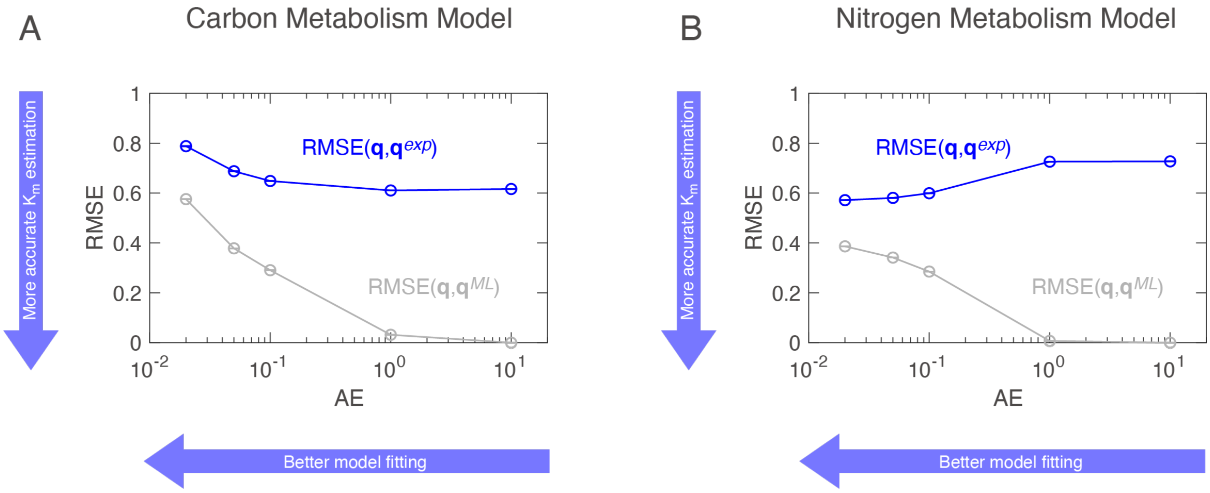

Figure S3. AE versus RMSE

(A) Carbon metabolism model. (B) Nitrogen metabolism model. AE indicates the allowable error for the badness-of-fit (BOF). RMSE($\mathbf{q},\mathbf{q}^{exp}$) indicates the RMSE between the K_m values estimated by the MLAGO and measured K_m values. RMSE($\mathbf{q}$, $\mathbf{q}^{ML}$) indicates the RMSE between the K_m values estimated by the MLAGO and machine learning-predicted K_m values. The circles represent mean values, and error bars represent ± standard deviation (n = 5). The error bars are not clearly visible because the standard deviation is small.
